# Evaluation of Thyroid Nodule Malignant Neoplasms and Obesity Among Children and Young Adults

**DOI:** 10.1001/jamanetworkopen.2021.16369

**Published:** 2021-07-09

**Authors:** Carlos A. Ortega, Jean-Nicolas Gallant, Sheau-Chiann Chen, Fei Ye, Huiying Wang, Ryan H. Belcher, Vivian L. Weiss

**Affiliations:** 1 Vanderbilt University School of Medicine, Nashville, Tennessee; 2 Department of Otolaryngology–Head and Neck Surgery, Vanderbilt University Medical Center, Nashville, Tennessee; 3 Department of Biostatistics, Vanderbilt University Medical Center, Nashville, Tennessee; 4 Department of Pathology, Microbiology, and Immunology, Vanderbilt University Medical Center, Nashville, Tennessee; 5 Monroe Carell Jr Children’s Hospital at Vanderbilt, Vanderbilt University Medical Center, Nashville, Tennessee

## Abstract

This cross-sectional study examines the association of obesity in childhood and adolescence with the incidence of malignant thyroid nodules.

## Introduction

While the prevalence of thyroid nodules in children, adolescents, and young adults in the United States has remained stable since the middle of the 20th century, the incidence of malignant nodules has been increasing among younger patients of all sexes, races, and ethnic groups. This trend cannot solely be explained by increased surveillance or improved detection because the diagnoses of small, early-stage and larger, late-stage thyroid cancers have both increased.^1^ Given that the rate of obesity in younger people in the United States has been increasing during this same period, we hypothesized that obesity may be associated with pediatric thyroid malignant neoplasms.

## Methods

All patients who underwent thyroid surgery at the Monroe Carell Junior Children’s Hospital at Vanderbilt University Medical Center (VUMC) between 2003 and 2019 were included in this retrospective cross-sectional study if they were younger than 21 years at the time of surgery. Approval was obtained from the VUMC institutional review board with a waiver of informed consent owing to the retrospective nature of this analysis. This cross-sectional study adheres to the Strengthening the Reporting of Observational Studies in Epidemiology (STROBE) reporting guideline. Patient demographic characteristics and clinical histories were extracted from the electronic health record. Self-identified patient race was recorded because of its potential confounding effect on socioeconomic status (SES). Thyroid nodule volumes were calculated based on ultrasound measurements and the ellipsoid formula ([length × width × depth] × [π / 6]). Household incomes were derived from US Census Bureau data. Body mass index *z* scores were calculated using the R package zscorer. All analyses were conducted in R version 4.0.2 (R Project for Statistical Computing) from March 2003 to July 2019. Statistical significance was set at *P* < .05, and all tests were 2-tailed. The eMethods in the Supplement contains more details regarding the modeling methods.

## Results

A total of 116 patients aged 21 years or younger (median [range] age, 16.1 [0.4 to 21.0] years; 93 [80%] female) underwent surgery for a thyroid nodule (Table). There was a significant difference in median (interquartile range) BMI *z* score between patients with a benign vs malignant nodule on final surgical pathology (0.7 [−0.2 to 1.4] vs 1.4 [0.4 to 2.3]; *P* = .02). There was no significant difference in other clinical (eg, history of thyroid disease) or socioeconomic (eg, household income, race) factors between the groups. Rather, as demonstrated by logistic regression, increases in BMI *z* score were significantly and independently associated with the probability of nodule malignant neoplasms at presentation (odds ratio, 1.551; 95% CI, 1.212-2.145; *P* = .008) (Figure). Importantly, patients with a higher BMI *z* score were not necessarily concealing a bigger nodule (Spearman *r* = 0.135; *P* = .24) or more extensive disease in their neck (*r* = 0.197, *P* = .33) at presentation.

**Table.** Patient Characteristics

Characteristic	Patients, No. (%)^a^			*P* value
	All (N = 116)	With benign nodules (n = 61)	With malignant nodules (n = 55)	
Age at surgery, median (IQR), y	16.1 (13.9 to 17.2)	16.3 (14.2 to 17.6)	15.9 (13.2 to 17.1)	.14
Sex				
Female	93 (80)	50 (82)	43 (78)	.61
Male	23 (20)	11 (18)	12 (22)	
Race				
White	101 (87)	53 (87)	48 (87)	.03
Black	8 (7)	6 (10)	2 (4)	
Hispanic	4 (3)	0	4 (7)	
Asian	2 (2)	2 (3)	0	
Middle Eastern	1 (1)	0	1 (2)	
Race				
Black, Hispanic, Asian, or other	15 (13)	8 (13)	7 (13)	.95
White	101 (87)	53 (87)	48 (87)	
BMI z score, median (IQR)	0.9 (0.0 to 2.0)	0.7 (−0.2 to 1.4)	1.4 (0.4 to 2.3)	.02
Dominant nodule volume, median (IQR), cm^3^	4.0 (1.3 to 8.9)	4.6 (1.9 to 8.6)	3.4 (1.3 to 9.3)	.78
History of thyroid disease				
Yes	36 (31)	16 (26)	20 (36)	.24
No	80 (69)	45 (74)	35 (64)	
Family history of history of thyroid cancer				
Yes	19 (16)	11 (18)	8 (15)	.61
No	97 (84)	50 (82)	47 (85)	
Household income, median (IQR), $^b^	52 964 (42 727 to 62 934)	49 952 (43 211 to 63 178)	54 811 (42 649 to 62 179)	.57
Insurance^c^				
Private	84 (73)	44 (73)	40 (73)	.94
Public	31 (27)	16 (27)	15 (27)	
Home smoke exposure^d^				
Yes	26 (27)	13 (26)	13 (28)	.90
No	70 (73)	36 (73)	34 (72)	

Abbreviations: BMI, body mass index; IQR, interquartile range.

^a^ Benign nodules included follicular adenoma and multinodular goiter; malignant nodules included papillary thyroid carcinoma (including variants), follicular thyroid carcinoma (and variants), Hürthle cell carcinoma, poorly differentiated carcinoma, and noninvasive follicular thyroid neoplasm with papillary-like nuclear features.

^b^ Household income was derived from the patient’s address and the median county income per the US Census Bureau.

^c^ Insurance was defined as public when the patient’s only insurance provider was TennCare (or Medicaid equivalent).

^d^ Home smoke exposure was defined as any individual who smokes in the home.

**Figure.**
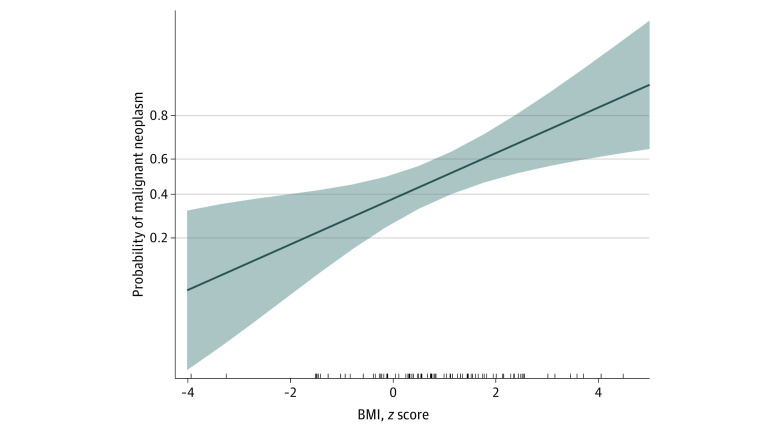
Association Between Body Mass Index (BMI) *z* Score, Socioeconomic Factors, and Risk of Malignant Neoplasms in Pediatric Thyroid Nodule Association of BMI *z* score on the probability of nodule malignant neoplasms, controlled for socioeconomic status, age, and race. Each tick on the x-axis indicates a case.

## Discussion

Obesity has been correlated with increased incidence for most types of cancer in adults, including thyroid cancer.^2^ In this study, we found that higher BMI *z* score was associated with an increased risk of thyroid nodule malignant neoplasms in individuals aged 21 years or younger. We used the BMI *z* score because obesity in childhood and adolescence is defined as an age-and sex-specific BMI in the 95th percentile or greater (BMI *z* score >1.65).^3^ While our study was limited by its retrospective cross-sectional nature and moderate sample size, we rigorously controlled for multiple variables that are often associated with obesity or SES. However, unlike among adults, we found that lower SES and race were not associated with an increased risk of thyroid nodule malignant neoplasms in this younger population. Likewise, neither BMI nor SES (data not shown) correlated with extensive disease at presentation in children.^4^ Taken together, these findings support the growing evidence that pediatric thyroid cancer has a different disease process than its adult counterpart.

Surgical pathology remains the criterion standard for assessing thyroid nodule malignant neoplasms. However, thyroidectomy is associated with higher rates of complication in children and adolescents than in adults.^5^ Guidelines suggest that surgical decision-making in younger patients may be informed by clinical factors that alter the risk of malignant neoplasms.^6^ Our study suggests that BMI *z* score should be considered such a clinical factor and considered when making the decision to proceed with surgery for a thyroid nodule in a younger patient.

## Notes

Supplement. **eMethods.** Supplementary Methods
