# Supplementary material for: Evaluation of Thyroid Nodule Malignant Neoplasms and Obesity Among Children and Young Adults
Source: JAMA Netw Open. 2021 Jul 9;4(7):e2116369. doi: 10.1001/jamanetworkopen.2021.16369 (PMC8271420; doi:10.1001/jamanetworkopen.2021.16369)
Supplement: Supplement. — eMethods. Supplementary Methods [file jamanetwopen-e2116369-s001.pdf]

Supplemental Online Content

Ortega CA, Gallant JN, Chen SC, et al. Evaluation of thyroid nodule malignant neoplasms and obesity among children and young adults. *JAMA Netw Open.* 2021;4(7):e2116369. doi:10.1001/jamanetworkopen.2021.16369

eMethods. Supplementary Methods

This supplemental material has been provided by the authors to give readers additional information about their work.

© 2021 Ortega CA et al. *JAMA Network Open.*

eMethods. Supplementary Methods

It was pre-determined that multiple imputation would be performed for covariate data with < 20% missing values. BMI z-scores (BMIz), race, age, and socioeconomic status (SES) variables—such as insurance type, smoke exposure, and median household income—were evaluated as potential predictors of thyroid nodule malignancy using logistic regressions. A multivariable linear regression model was fit to assess the associations between BMIz and nodule size, adjusted for the same set of covariates. BMIz was initially assumed to have a nonlinear relationship with the outcomes and modeled using a restricted cubic spline function with 3 knots. The nonlinearity assumption of BMIz was tested non-significant we therefore included BMIz as a linear term in the final models. All analyses were performed using R version 4.0.2.

© 2021 Ortega CA et al. *JAMA Network Open.*
